# Description and Prognosis of Patients with Recovered Dilated Cardiomyopathy: A Retrospective Cohort Study

**DOI:** 10.31083/j.rcm2507246

**Published:** 2024-07-04

**Authors:** Pengda Li, Cunhang Jia, Ning Sun, Junyong Zhao, Zelan Wang, Wenjian Luo, Zebi Wang, Shaofa Wu, Ling Chen, Xiaolin Luo, Shulin Ou, Xi Liu, Zhexue Qin

**Affiliations:** ^1^Department of Cardiology, Xinqiao Hospital, Army Medical University (Third Military Medical University), 400037 Chongqing, China; ^2^Department of Cardiology, People’s Hospital of Nanchuan District, 408400 Chongqing, China

**Keywords:** recovered dilated cardiomyopathy, relapse, clinical description, prognosis

## Abstract

**Background:**

With the recent advances in the treatment of heart failure (HF), it is intriguing that a very small number of patients with dilated cardiomyopathy (DCM) have been observed as being fully recovered. However, knowledge of the progression and prognosis of patients with recovered DCM remains sparse. Herein, we conducted this study to investigate the clinical characteristics and prognosis of patients with recovered DCM.

**Methods:**

Consecutive patients with recovered DCM referred to our hospital between March 2009 and May 2021 were included. The recovered DCM patients were categorized into relapse and non-relapse groups. The primary endpoint was all-cause death, and the secondary endpoint was HF re-hospitalization during follow-up. Multivariate analyses were performed to identify predictors of relapse among recovered DCM patients. Kaplan–Meier analyses were used to assess the prognostic significance of relapse.

**Results:**

A comparatively large cohort of 122 recovered DCM patients from 10,029 DCM patients was analyzed. During a median follow-up duration of 53.5 months, the relapse rate among recovered DCM patients was 15.6% (19/122). Age (odds ratio, OR 1.079, 95% confidence interval, CI: 1.014–1.148; *p* = 0.017), systolic blood pressure (SBP) at diagnosis (OR 0.948, 95% CI: 0.908–0.990; *p* = 0.015) and changes in left ventricular ejection fraction from diagnosis to recovery (ΔLVEF) (OR 0.898, 95% CI: 0.825–0.978; *p* = 0.013) were identified as predictors of relapse. Furthermore, among 122 patients, 5 (4.1%) experienced death, and 12 (9.8%) underwent HF re-hospitalization. Four deaths occurred in the relapse group, with one in the non-relapse group. All deaths were attributed to cardiovascular events. The long-term prognosis of the relapse group was significantly worse compared to the non-relapse group by Kaplan–Meier analysis (*p* < 0.001 based on the log-rank test). Multivariate analyses significantly associated relapse with all-cause mortality in recovered DCM patients (hazard ratio, HR 7.738, 95% CI: 1.892–31.636; *p* = 0.004).

**Conclusions:**

Recovered DCM patients are at risk of relapse. Older age, lower SBP, and smaller ΔLVEF were independently associated with relapse in recovered DCM patients. Relapse after recovery was related to an unfavorable long-term prognosis.

## 1. Introduction

Dilated cardiomyopathy (DCM) is a myocardial disease characterized by left ventricular systolic dysfunction and dilatation, often leading to heart failure (HF), other adverse cardiovascular events, and even death [1, 2]. In the past, the long-term prognosis of DCM was less favorable, with a 10-year mortality rate of up to 40% [3]. However, advancements in pharmacologic therapy and understanding of disease progression have resulted in some DCM patients experiencing improved cardiac function [4, 5, 6, 7, 8]. Despite numerous studies on DCM patients after improved cardiac function [9, 10], limited research has focused on the prognosis of recovered DCM patients. Consequently, the progression and prognosis of recovered DCM patients remain sparse.

Recovered DCM is defined as DCM where symptoms, cardiac structure, and function are completely recovered [11]. To our knowledge, no cohort studies are currently related to a completely recovered DCM. Moreover, recovered DCM patients are the subgroup with better treatment responsiveness, namely left ventricular reverse remodeling (LVRR). LVRR involves a reduction in dimensions, normalization of shape, and substantial improvements in both systolic and diastolic function [12]. Previous studies have explored the prevalence of LVRR, identified laboratory and clinical risk factors of LVRR, and assessed its prognostic role during long-term follow-up in DCM patients [10, 13, 14]. Previous studies suggested that LVRR is a significant treatment objective for DCM patients and indicates a better prognosis [13]. However, there is limited literature specifically focusing on recovered DCM patients, particularly with regard to laboratory and clinical predictors of relapse and its impact on prognosis. Investigating this subgroup of DCM patients after recovery could be of significant value for long-term risk stratification, providing prognostic insights and facilitating individualized clinical management.

Therefore, this study aimed to determine the prevalence of relapse and features of recovered DCM patients, uncover the predictive clinical parameters for DCM relapse, and, finally, compare the prognosis of recovered DCM patients with and without relapse.

## 2. Materials and Methods

### 2.1 Study Design

This study was designed as an observational, retrospective cohort study conducted at Xinqiao Hospital, Army Medical University, Chongqing, China. Patients were systematically evaluated using medical records, relevant examinations, and medical treatments according to the latest guidelines [15]. Demographic information, physical examinations, results of routine blood tests, serial echocardiographic studies, and electrocardiograms were collected from medical records during enrollment and follow-up. For this study, included recovered DCM patients were classified into either the relapse or non-relapse group.

This study was in accordance with the principles of the Declaration of Helsinki. The ethics committee of Xinqiao Hospital of Army Medical University reviewed and approved this study (No. 2023-089-01), which allowed retrospective review of medical records and waived the need for informed consent.

### 2.2 Study Population

All consecutive patients with DCM referring to Xinqiao Hospital of Army Medical University between March 2009 and May 2021 were screened. DCM is defined as the presence of left ventricular (LV) dilatation and global or regional systolic dysfunction that cannot be explained solely by abnormal loading conditions (e.g., hypertension, valve disease, congenital heart disease) or coronary artery disease according to current criteria [16]. This study included patients diagnosed with DCM who had undergone at least one echocardiographic evaluation in addition to the baseline during the follow-up period. Subsequently, recovered DCM patients were identified through echocardiographic evaluation, with agreement from two cardiologists.

### 2.3 Definition of Recovered DCM and Relapse

According to current consensus, the definition of recovered DCM includes the combined presence of the following at follow-up: (1) N-terminal pro-B-type natriuretic peptide (NT-pro-BNP) concentration less than 250 ng/L and currently asymptomatic, (2) an increase in left ventricular ejection fraction (LVEF) of at least 10 points or a follow-up LVEF ≥50%, when LVEF was 45% to 49% at enrollment, (3) a decrease in indexed left ventricular end-diastolic diameter (LVEDDI) of at least 10% or LVEDDI ≤33 ${\mathrm{mm/m}}^{2}$ [11]. Moreover, patients with concomitant pericardial disease, congenital heart disease, acute coronary syndrome, persistent supraventricular tachyarrhythmias, cor pulmonale, advanced systemic disease affecting short-term prognosis, severe organic valve disease, or stress cardiomyopathy [17], were excluded [18]. Patients with DCM who did not undergo follow-up echocardiography were also excluded, as the occurrence of recovery could not be determined.

Relapse of DCM was defined by meeting at least one of the following criteria: A reduction in LVEF by more than 10% and to less than 50%; an increase in LVEDDI by more than 10% and higher than the normal range; a two-fold rise in baseline NT-pro-BNP concentration and greater than 400 ng/L; or clinical evidence of HF based on signs and symptoms assessed [11]. Related variables in the relapse group were compared to the non-relapse group with emphasis. Changes in related variables after relapse compared to baseline were also evaluated. Without the occurrence of atrial fibrillation, NT-pro-BNP was converted to BNP using the formula BNP = NT-pro-BNP/5.75, whereas, in cases of atrial fibrillation, the conversion was performed using the formula BNP = NT-pro-BNP/8.03 [19].

### 2.4 Endpoints

The primary endpoint was defined as all-cause death. The secondary endpoint was defined as HF re-hospitalization during the follow-up period. The endpoint information was obtained directly from medical records or patients and their guardians.

### 2.5 Statistical Analysis

Variables are presented as frequencies (percentage), mean ± standard deviation (SD), and median (interquartile range (IQR)), as appropriate. Differences between patients with and without relapse were tested using chi-squared tests (or Fisher’s exact test when necessary) for categorical variables and *t*-tests or the non-parametric Mann–Whitney U test for continuous variables, as necessary. Related variables at follow-up were compared between patients who relapsed and those who did not.

To identify factors associated with relapse among recovered DCM patients using baseline and follow-up variables, a univariate screening of relevant parameters was conducted to estimate univariable logistic regression models. Selected parameters from the univariate analysis were then used to estimate the multivariable logistic regression equation. Kaplan–Meier analysis was used to calculate survival curves, and the log-rank test was performed to compare survival curves. Similarly, univariate and multivariate Cox proportional hazard regression analyses were performed to assess covariates associated with event-free survival.

Statistical significance was defined as *p* < 0.05. The analyses were conducted using SPSS version 25.0 (IBM Corporation, Armonk, NY, USA) software.

## 3. Results

### 3.1 Baseline Characteristics

**Fig. 1.**
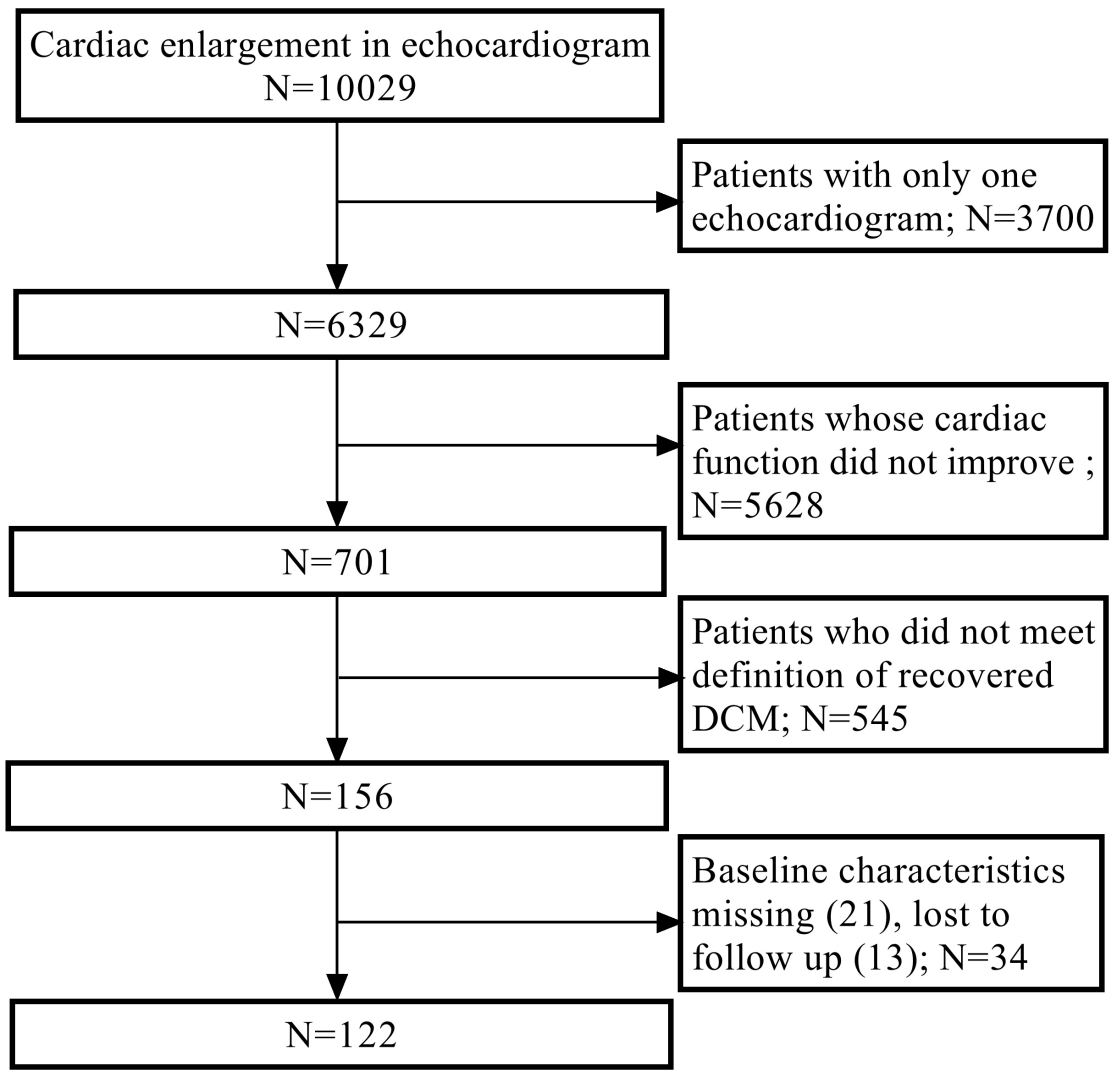
**Study flow chart of enrollment.** DCM, dilated cardiomyopathy.

A comparatively large cohort of 122 recovered DCM patients based on 10,029 DCM patients was analyzed according to the specified inclusion and exclusion criteria (Fig. 1). Among these patients, 19 (15.6%) experienced relapses following recovery.

The baseline characteristics of the patients are shown in Table 1. Overall, the median age was 55 years at enrollment, and 71.3% of the patients were males. Hypertension was reported in 46.7% of the patients, with chronic obstructive pulmonary disease (COPD) in 4.9%, diabetes mellitus in 12.3%, atrial fibrillation (AF) in 8.3%, New York Heart Association (NYHA) functional class III–IV in 78.7%, and left bundle branch block (LBBB) in 9.0% of the study population. At inclusion, the median LVEF was 36%, median LVEDDI was 34 ${\mathrm{mm/m}}^{2}$, and median NT-pro-BNP levels were 4286.6 ng/L. The majority of patients received beta-blockers, angiotensin-converting enzyme inhibitors or angiotensin receptor blockers (ACEI/ARB), and mineralocorticoid antagonists (MAR) as treatment (25.4% and 22.1% were treated with angiotensin receptor–neprilysin inhibitor (ARNI) and digoxin, respectively), while cardiac resynchronization therapy (CRT) was implemented in 9% of the patients.

**Table 1.** **Baseline characteristics at diagnosis**.

		Overall (122)	Non-relapse (103)	Relapse (19)	*p* value
Age (years)		55 (48, 62)	55 (47, 59)	61 (53, 70)	0.005
Male gender, n (%)		87 (71.3)	74 (71.8)	13 (68.4)	0.762
BMI		24.9 (22.2, 26.7)	24.8 (22.4, 26.9)	25.0 (20.5, 25.6)	0.244
Hypertension, n (%)		57 (46.7)	52 (50.2)	5 (26.3)	0.052
Diabetes mellitus, n (%)		15 (12.3)	13 (12.6)	2 (10.5)	1.000
COPD, n (%)		6 (4.9)	2 (1.9)	4 (21.1)	0.005
Hyperlipidemia, n (%)		17 (13.9)	15 (14.6)	2 (10.5)	0.915
Tachycardia, n (%)		41 (34.2)	36 (35.6)	5 (26.3)	0.432
Bradycardia, n (%)		3 (2.5)	2 (2.0)	1 (5.3)	0.407
HR (bpm)		93 (80, 122)	96 (80, 114)	82 (72, 112)	0.199
SBP (mmHg)		128 (115, 141)	130 (117, 145)	116 (100, 126)	0.002
DBP (mmHg)		80 (70, 99)	80 (70, 100)	78 (70, 80)	0.063
III–IV NYHA class, n (%)		96 (78.7)	79 (79)	17 (89.5)	0.465
AF, n (%)		10 (8.3)	10 (9.8)	0 (0)	0.331
LBBB, n (%)		11 (9.0)	9 (8.7)	2 (10.5)	1.000
QRS length (ms)		102.3 ± 26.0	99.7 ± 24.3	113.7 ± 31.0	0.093
LVEF		36 (33, 39)	33 (26, 36)	34 (28, 36)	0.506
LVEDDI (${\mathrm{mm/m}}^{2}$)		34.0 (31.7, 38.6)	34.0 (31.2, 38.6)	35.0 (33.0, 40.3)	0.328
NT-pro-BNP (ng/L)		4286.6 (2634.9, 6195.6)	4370.0 (2823.3, 6713.1)	3852.5 (2363.3, 5787.4)	0.279
Hb (g/L)		145 (133, 157)	149 (135, 158)	136 (129, 145)	0.051
Treatment					
	Digoxin, n (%)	27 (22.1)	19 (18.4)	8 (42.1)	0.047
	Beta-blockers, n (%)	83 (68.0)	72 (69.9)	11 (57.9)	0.302
	Loop diuretics, n (%)	77 (63.1)	65 (63.1)	12 (63.2)	0.997
	MRA, n (%)	92 (75.4)	77 (74.8)	15 (78.9)	0.920
	ACEI/ARB, n (%)	92 (75.4)	79 (76.7)	13 (68.4)	0.631
	ARNI, n (%)	31 (25.4)	25 (24.3)	6 (31.6)	0.700
	ICD, n (%)	2 (1.6)	1 (1.0)	1 (5.3)	0.176
	CRT, n (%)	11 (9.0)	8 (7.8)	3 (15.8)	0.493

Data presented as frequencies (percentage) for categorical variables and median (IQR) for continuous variables. BMI, body mass index; HR, heart rate; SBP, systolic blood pressure; DBP, diastolic blood pressure; COPD, chronic obstructive pulmonary disease; NYHA, New York Heart Association; AF, atrial fibrillation; LBBB, left bundle branch block; LVEF, left ventricle ejection fraction; LVEDDI, left ventricular end-diastolic diameter index; NT-pro-BNP, N-terminal pro-B-type natriuretic peptide; Hb, hemoglobin; MRA, mineralocorticoid receptor antagonist; ACEI/ARB, angiotensin-converting enzyme inhibitors/angiotensin II receptor antagonists; ARNI, angiotensin receptor–neprilysin inhibitor; ICD, implantable cardioverter-defibrillator; CRT, cardiac resynchronization therapy; IQR, interquartile range.

In contrast with the non-relapse group, the relapse group had a significantly older age, a higher incidence of COPD, and greater use of digoxin (*p* < 0.05 for all). Conversely, the systolic blood pressure (SBP) levels at diagnosis (*p* = 0.002) were significantly lower in the relapse group. No significant statistical differences were observed for the other variables. Notably, no significant difference was observed in ARNI use between the two groups.

### 3.2 Follow-Up

Clinical, electrocardiogram, and echocardiography data were analyzed during follow-up after recovery for patients with DCM. The relevant variables are shown in Table 2. It is worth noting that none of the DCM patients in our cohort were classified as NYHA functional class IV after recovery. Moreover, after recovery, in the relapse group, there was a higher incidence of NYHA functional class III (*p* = 0.001), medication withdrawal (*p* = 0.003), and LVEDDI levels (*p* = 0.012), while SBP (*p* = 0.045) and diastolic blood pressure (DBP) levels (*p* = 0.012) were lower compared to the non-relapse group. Other variable comparisons did not show statistically significant differences.

**Table 2.** **Patients’ characteristics after DCM recovery**.

	Overall (122)	Non-relapse (103)	Relapse (19)	*p* value
HR (bpm)	80 (77, 88)	80 (71, 87)	78 (69, 92)	0.945
SBP (mmHg)	127 (118, 132)	129 (120, 133)	118 (99, 127)	0.045
DBP (mmHg)	80 (70, 86)	80 (71, 86)	70 (65, 76)	0.012
NYHA class III, n (%)	4 (3.3)	1 (1.0)	3 (15.8)	0.001
LBBB, n (%)	6 (4.9)	5 (4.9)	1 (5.3)	0.295
QRS length (ms)	92 (86, 104)	90 (86, 101)	104 (81, 114)	0.324
LVEF	61 (58, 65)	62 (58, 65)	59 (57, 62)	0.071
LVEDDI (${\mathrm{mm/m}}^{2}$)	26.0 (24.9, 29.0)	25.9 (24.7, 28.8)	28.4 (26.8, 30.4)	0.012
NT-pro-BNP after recovery	196.1 (114.4, 230.0)	181.4 (109.2, 229.9)	218.5 (166.7, 247.2)	0.085
Recovery time after treatment (months)	16 (9, 29)	15.5 (9, 30)	21 (10, 26)	0.839
Regular medication, n (%)	96 (78.7)	83 (80.6)	13 (68.4)	0.376
Medication withdrawal, n (%)	24 (19.7)	15 (14.6)	9 (47.4)	0.003
Relapse time after recovery (months)			23 (17, 45)	

Data presented as frequencies (percentage) for categorical variables and median (IQR) for continuous variables. HR, heart rate; SBP, systolic blood pressure; DBP, diastolic blood pressure; NYHA, New York Heart Association; LBBB, left bundle branch block; LVEF, left ventricle ejection fraction; LVEDDI, left ventricular end-diastolic diameter index; NT-pro-BNP, N-terminal pro-B-type natriuretic peptide; IQR, interquartile range.

**Fig. 2.**
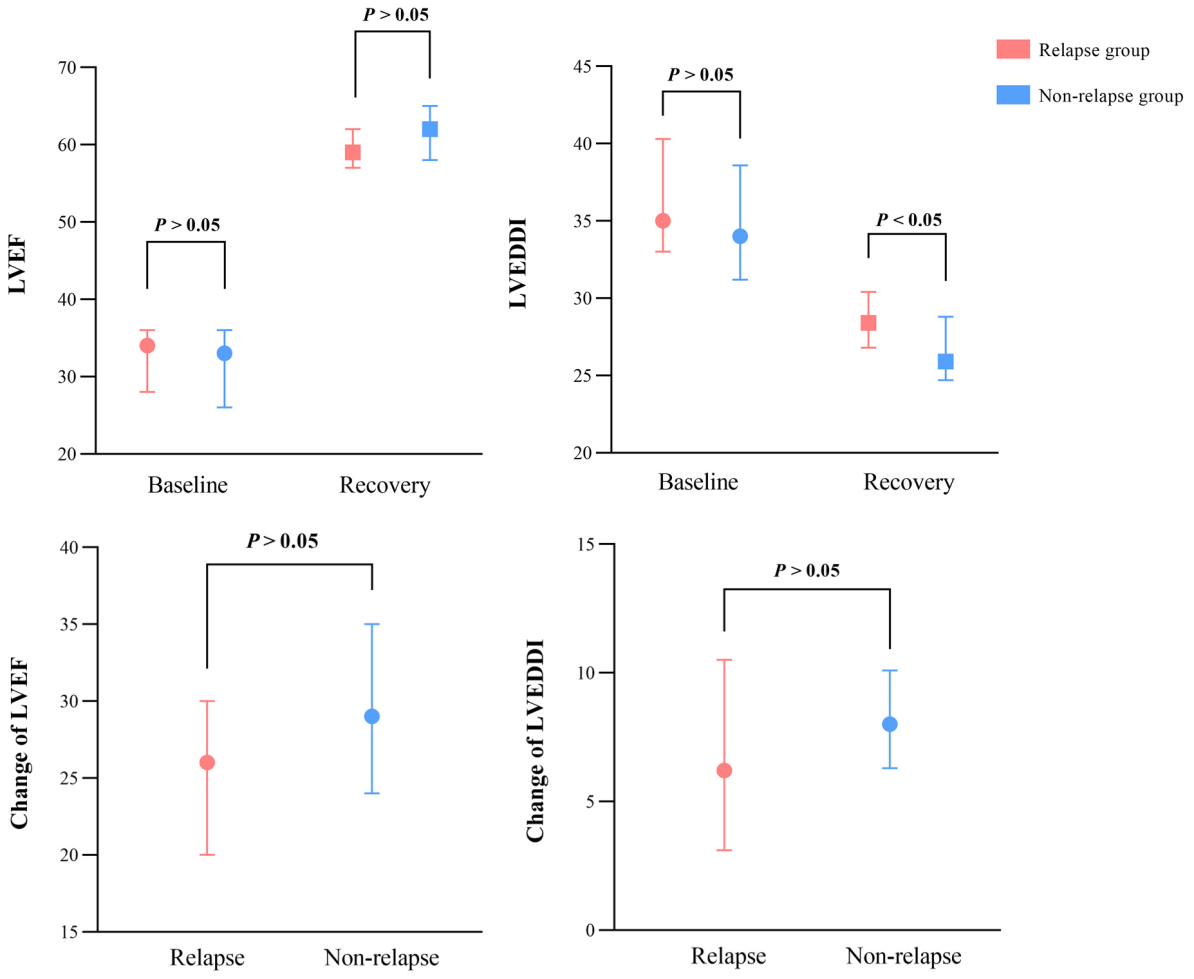
**Comparison of LVEF, LVEDDI, and their change from diagnosis to recovery between relapse and non-relapse groups.** LVEF, left ventricular ejection fraction; LVEDDI, left ventricular end-diastolic diameter.

In addition, the changes in LVEF and LVEDDI from diagnosis to recovery were compared between groups (Fig. 2; **Supplementary Table 1**). The median LVEF and LVEDDI at diagnosis were not significantly different between the relapse and non-relapse groups (*p* > 0.05). During follow-up, the relapse group exhibited a marked enlargement in LVEDDI (*p* = 0.012). However, the changes in left ventricular dimensions and LVEF between groups were comparable, although not statistically significantly different (*p* > 0.05).

### 3.3 Predictors of Relapse in Recovered DCM Patients

**Fig. 3.**
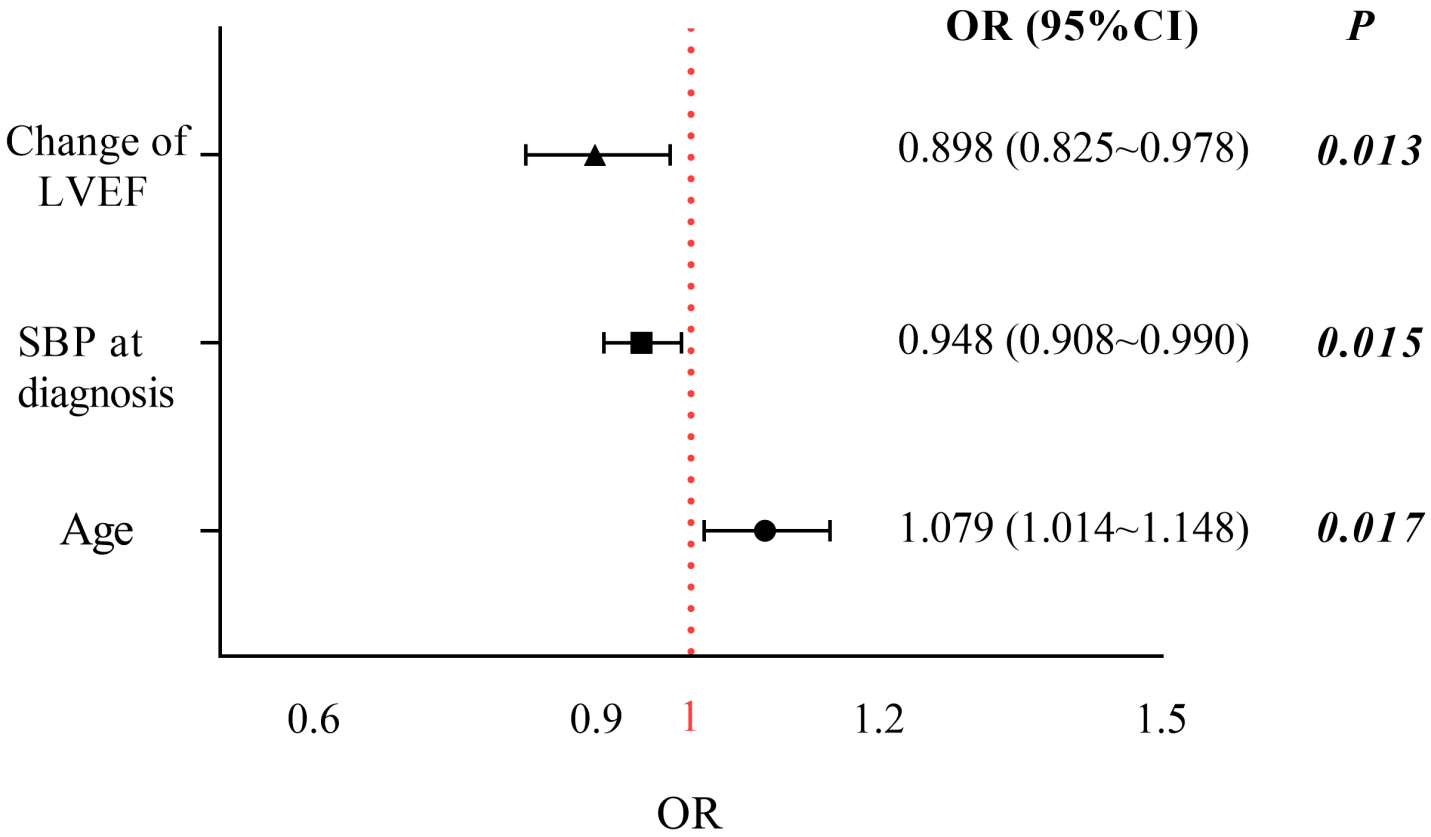
**Significant relapse-associated clinical variables at multivariate logistic regression analysis (*p* < 0.05)**. The model adjusted for age, COPD, SBP, digoxin, III–IV NYHA class after recovery, medication withdrawal, LVEDDI after recovery, and ΔLVEF. CI, confidence interval; OR, odds ratio; LVEF, left ventricle ejection fraction; SBP, systolic blood pressure; COPD, chronic obstructive pulmonary disease; NYHA, New York Heart Association; LVEDDI, left ventricular end-diastolic diameter index.

Logistic regression analyses were performed to identify predictors of relapse in recovered DCM patients (Fig. 3; **Supplementary Table 2**). In the univariate analysis, the age, SBP at diagnosis, use of digoxin, incidence of COPD, NYHA functional class III after recovery, medication withdrawal, LVEDDI after recovery, and ΔLVEF were found to be significantly related to relapse. However, only the age (OR 1.079, 95% CI: 1.014–1.148; *p* = 0.017), SBP at diagnosis (OR 0.948, 95% CI: 0.908–0.990; *p* = 0.015), and ΔLVEF (odds ratio, OR 0.898, 95% confidence interval, CI: 0.825–0.978; *p* = 0.013) were identified as independent risk predictors in the multivariate analysis.

### 3.4 Relapse and Endpoints

Following a median follow-up of 53.5 months, 19 (15.6%) patients experienced relapse, and 5 (4.1%) deaths occurred among the 122 patients (**Supplementary Fig. 1**). Among those who died, four relapsed after recovery. All deaths were attributed to cardiovascular events. Additionally, the re-hospitalization rate for HF was 9.8% (12/122).

**Fig. 4.**
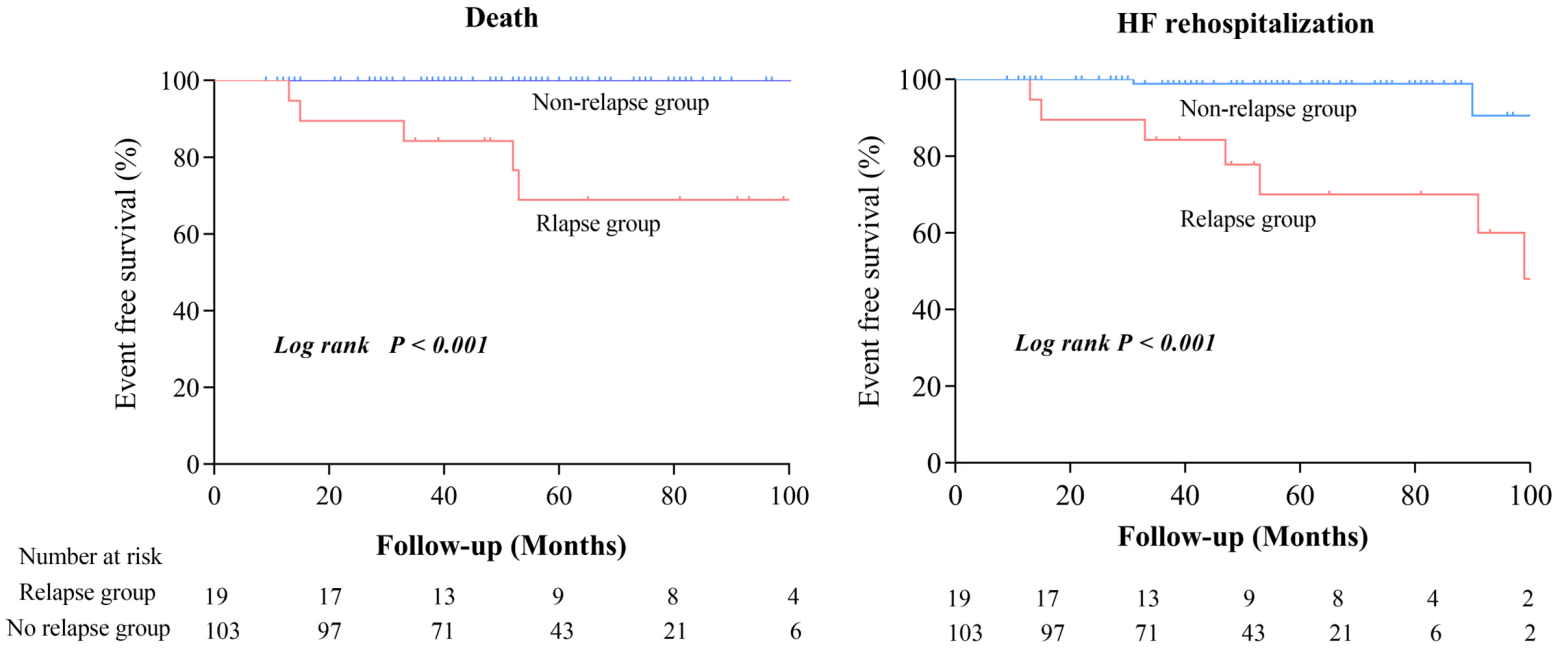
**Long-term outcome of recovered dilated cardiomyopathy patients.** HF, heart failure.

The survival curves based on the Kaplan–Meier analysis demonstrated that the long-term prognosis of the relapse group was significantly worse compared to the non-relapse group (*p* < 0.001 based on the log-rank test) (Fig. 4).

In the univariate Cox regression analyses, relapse after recovery, incidence of COPD, as well as NYHA functional class III after recovery were significantly related to the composite endpoint of death and HF re-hospitalization (Table 3). Moreover, in the multivariate analyses, relapse after recovery (hazard ratio, HR 7.738, 95% CI: 1.892–31.636; *p* = 0.004) was found to be an independent risk factor predicting the composite endpoint, regardless of other relevant parameters.

**Table 3.** **Factors associated with death/HF re-hospitalization in dilated cardiomyopathy patients after recovery**.

Variable	Univariate analysis			Multivariate analysis		
	HR	95% CI	*p*	HR	95% CI	*p*
Relapse after recovery	10.099	2.645–38.564	0.001	7.738	1.892–31.636	0.004
COPD, n (%)	8.077	1.960–33.290	0.004			
NYHA class III after recovery	10.261	1.938–54.312	0.006			

Results presented as HRs and 95% CIs from the Cox proportional hazards regression models. CI, confidence interval; HR, hazard ratio; COPD, chronic obstructive pulmonary disease; NYHA, New York Heart Association.

## 4. Discussion

Treatment-induced cardiac recovery has recently been considered an important prognostic predictor in managing patients with DCM [20]. Over the past decade, several studies have suggested that a significant proportion of DCM patients (approximately 40%) can experience cardiac reverse remodeling, specifically referring to LVRR [21, 22, 23, 24]. Therefore, some studies have begun to focus on the clinical description and prognostic value of LVRR [22, 23, 24]. However, as the subgroup with better treatment responsiveness among DCM patients with LVRR, certain aspects of recovered DCM patients, such as the rate and risk of recurrent LV dysfunction (relapse after recovery) and its impact on prognosis, remain obscure.

In this study, we retrospectively analyzed the clinical description, predictors of relapse, and prognosis of patients with recovered DCM within a median follow-up period of 53.5 months. The main findings were as follows: (1) the incidence of relapse in recovered DCM patients over long-term follow-up was 15.6%; (2) age, SBP, and ΔLVEF were found to be independent risk factors predicting relapse. These factors facilitated early identification and risk stratification for clinical management; (3) the relapse group, who had a higher risk of death and HF re-hospitalization, had a worse prognosis compared to the non-relapse group.

To our knowledge, the term ventricular remodeling refers to a change in ventricular architecture with an associated increase in volume and abnormal chamber configuration [25]. Furthermore, ventricular reverse remodeling can occur after myocardial injury and when wall stress is reduced [26]. However, there is currently no consensus on the definition of recovered DCM. The TRED-HF study, published in the *Lancet* in 2019, a high-quality randomized controlled study, defines this concept by combining echocardiographic and NT-pro-BNP symptoms and signs [11]. In this study, we adopted the most commonly used definition of recovered DCM to minimize selection bias due to variability in definitions.

As the risk of relapse in recovered DCM patients and its influence on prognosis have been obscure, there is currently no clear definition. Therefore, regarding the definition of relapse after recovery in DCM patients, we also referred to the criteria of the recently conducted TRED-HF study [11]. In that study, patients with DCM demonstrated a relapse rate exceeding 40% in the arm of treatment withdrawal. Additionally, based on LVEF alone, some studies reported a recurrent LV dysfunction rate of 20–41% during follow-up in patients with DCM despite previous LVEF normalization [18, 27]. In our study, we documented a 15.6% rate of relapse after recovery in patients with DCM. This rate has not been reported yet, which may provide a reference for subsequent related research. Meanwhile, due to the limitations of retrospective research, the relapse rate found in our study needs further confirmation through similar studies.

Most previous studies have analyzed factors associated with improved cardiac function in patients with DCM [10, 13, 14, 21, 22, 23]. However, to the best of our knowledge, there is no cohort study on the long-term prognosis for DCM after recovery in a real-world setting. Based on this, we analyzed factors associated with relapse in recovered DCM patients. Our study found that older age, lower SBP at diagnosis, and smaller ΔLVEF from baseline to recovery were associated with a greater risk of disease recurrence. These results were less consistent with previous studies on factors related to LVRR. Marco Merlo *et al*. [10] reported that higher baseline SBP and the absence of left bundle branch block (LBBB) were independent predictors of LVRR. Carles Díez-López *et al*. [14] also suggested that the predictors of LVRR included shorter duration of HF, alcoholic cardiomyopathy, absence of LBBB, and lower baseline LVEF and NT-pro-BNP levels. Another noteworthy point is the role of digoxin in influencing relapse incidence. It was reported that the relapse group had a significantly greater use of digoxin compared to the non-relapse group in our study. It is well known that previous studies have shown that digoxin does not reduce overall mortality, although it reduces overall hospitalization rates and hospitalization rates for worsening heart failure [28, 29]. We speculate that the relapse group patients had more severe and urgent symptoms at the time of diagnosis, such as severe inadequate tissue and organ perfusion, which increased their likelihood of using digoxin. These phenomena may suggest that the mechanisms linking recovery to relapse in recovered DCM patients are not as consistent as previously thought, and there may still be additional aspects for us to explore. Therefore, the preliminary findings of this observational study should be further explored and validated in the future.

The prediction of prognosis in patients diagnosed with recovered DCM poses significant challenges, and the findings from our study hold substantial implications for the clinical management of affected individuals. Our study revealed that relapse in recovery was an independent risk factor for prognosis in patients with DCM. This finding, although easy to understand, was further confirmed by us. Many previous studies have proved poorer long-term prognoses in DCM patients without LVRR [10, 12, 14, 22], although they have not further investigated the association between the relapse after recovery and long-term prognoses. Unfortunately, the long-term prognostic analysis in our study did not identify other factors related to the endpoints besides the relapse, which may be attributed to the biases in retrospective studies, suggesting the need for more appropriate endpoint definitions.

The present study had several limitations worth mentioning. Firstly, the retrospective design introduced common biases associated with observational studies. The study population was enrolled at a single center, limiting the generalizability of the results. Secondly, although magnetic resonance imaging data might be more sensitive and specific for diagnosing improvement of cardiac function or relapse, they were not systematically available. However, we employed widely accepted diagnostic criteria based on echocardiography. Thirdly, some patients may have met our inclusion criteria but experienced only one echocardiographic evaluation. However, these patients were not enrolled according to the study’s criteria, which may have reduced the number of cases. Finally, blood pressure and heart rate data were mostly obtained through patient questioning, and accurate case records were lacking, which could introduce recall bias.

## 5. Conclusions

In recovered DCM patients, the relapse rate was approximately 16%, and relapse after recovery was related to a worse long-term prognosis. Older age and lower SBP at diagnosis, together with smaller ΔLVEF from diagnosis to recovery, were identified as clinical variables associated with relapse. These findings highlight the importance of deeper phenotyping of recovered DCM and emphasize the practicality of existing clinical parameters for effective patient management and prognostic analysis.

## Data Availability

The datasets used and/or analyzed during this study are available from the corresponding author upon reasonable request.

## Abbreviations

DCM, dilated cardiomyopathy; LVRR, left ventricular reverse remodeling; ACEI, angiotensin-converting enzyme inhibitor; ARB, angiotensin receptor blocker; ARNI, angiotensin receptor–neprilysin inhibitor; MRA, mineralocorticoid receptor antagonist; SGLT2, sodium–glucose cotransporter-2; RASS, renin-angiotensin-aldosterone; NYHA, New York Heart Association; LVEF, left ventricular ejection fraction; LVEDDI, indexed left ventricular end-diastolic diameter; HF, heart failure; COPD, chronic obstructive pulmonary disease; AF, atrial fibrillation; LBBB, left bundle branch block; SBP, systolic blood pressure; NT-pro-BNP, N-terminal pro-B-type natriuretic peptide; SD, standard deviation; IQR, interquartile range; HR, hazard ratio.

## Supplementary Material

Supplementary material associated with this article can be found, in the online version, at https://doi.org/10.31083/j.rcm2507246.
