# Supplementary material for: Description and Prognosis of Patients with Recovered Dilated Cardiomyopathy: A Retrospective Cohort Study
Source: Rev Cardiovasc Med. 2024 Jul 4;25(7):246. doi: 10.31083/j.rcm2507246 (PMC11317342; doi:10.31083/j.rcm2507246)
Supplement: Supplementary file 1 [file 2153-8174-25-7-246-s1.docx]

Supplementary Table 1. Changes in echocardiographic characteristics from diagnosis to recovery.

|  | Overall (122) | Non-relapse (103) | Relapse (19) | *P* value |
| --- | --- | --- | --- | --- |
| ***Diagnosis*** |  |  |  |  |
| LVEF (%) | 36 (33,39) | 33 (26,36) | 34 (28,36) | 0.506 |
| LVEDDI (mm/m^2^) | 34.0 (31.7, 38.6) | 34.0 (31.2, 38.6) | 35.0 (33.0, 40.3) | 0.328 |
| ***Recovery*** |  |  |  |  |
| LVEF (%) | 61 (58,65) | 62 (58,65) | 59 (57,62) | 0.071 |
| LVEDDI (mm/m^2^) | 26.0 (24.9,29.0) | 25.9 (24.7,28.8) | 28.4 (26.8,30.4) | 0.012 |
| ***Median change*** |  |  |  |  |
| LVEF (%) | 29 (23, 35) | 29 (24, 35) | 26 (20, 30) | 0.080 |
| LVEDDI (mm/m^2^) | 8.0 (6.0, 10.2) | 8.0 (6.3, 10.1) | 6.2 (3.1, 10.5) | 0.211 |

Data presented as number (%) for categorical variables, and as median (interquartile range) for continuous variables. LVEF, left ventricle ejection fraction; LVEDDI, left ventricular end-diastolic diameter index.

Supplementary Table 2. Factors associated with relapse in recovered DCM patients.

| **Variable** | **Univariate Analysis** | | | **Multivariate Analysis** | | |
| --- | --- | --- | --- | --- | --- | --- |
|  | **OR** | **95%CI** | ***P* value** | **OR** | **95%CI** | ***P* value** |
| Age (years) | 1.073 | 1.021-1.127 | 0.005 | 1.079 | 1.014-1.148 | 0.017 |
| COPD, n (%) | 13.467 | 2.267-80.009 | 0.004 |  |  |  |
| SBP at diagnosis (mmHg) | 0.954 | 0.923-0.985 | 0.004 | 0.948 | 0.908-0.990 | 0.015 |
| Digoxin | 3.215 | 1.139-9.078 | 0.027 |  |  |  |
| NYHA class III after recovery | 22.846 | 2.210-236.192 | 0.009 |  |  |  |
| Medication withdrawal | 5.280 | 1.840-15.147 | 0.002 |  |  |  |
| LVEDDI after recovery | 1.262 | 1.065-1.495 | 0.007 |  |  |  |
| △LVEF | 0.940 | 0.886-0.997 | 0.039 | 0.898 | 0.825-0.978 | 0.013 |

To predict relapse from baseline variables, first a univariate screening of all clinical-laboratory parameters of patients at enrollment was made (estimating univariable logistic regression models variable by variable); then to estimate the multivariable logistic regression equation, a stepwise backward conditional algorithm was applied to the list of selected parameters (i.e., with p < 0.1) at the univariate procedure. COPD, chronic obstructive pulmonary disease; SBP, systolic blood pressure; NYHA, New York Heart Association; LVEDDI, left ventricular end-diastolic diameter index; LVEF, left ventricle ejection fraction.


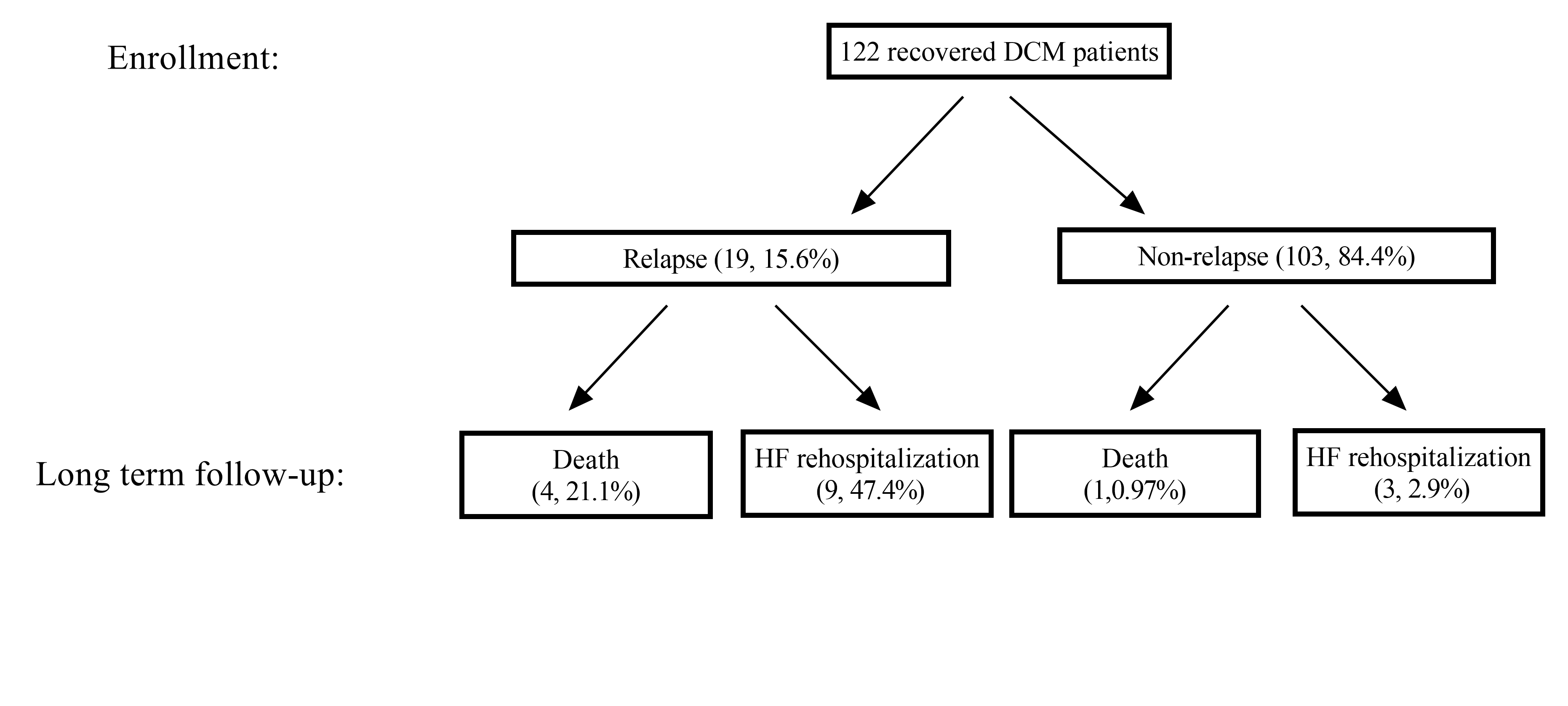


**Supplementary Fig. 1. Follow-up of recovered DCM study population.** DCM, dilated cardiomyopathy; HF, heart failure.
